# A Procedure for Analyzing Mandible Roto-Translation Induced by Mandibular Advancement Devices

**DOI:** 10.3390/ma13081826

**Published:** 2020-04-13

**Authors:** Giovanni Bruno, Alberto De Stefani, Edoardo Conte, Manila Caragiuli, Marco Mandolini, Daniele Landi, Antonio Gracco

**Affiliations:** 1 Department of Neuroscience, Section of Dentistry, University of Padua, 35100 Padua, Italy; alberto.de.stefani@hotmail.it (A.D.S.); edoardoconte.94@gmail.com (E.C.); antonio.gracco@unipd.it (A.G.); 2 Department of Industrial Engineering and Mathematical Sciences, Università Politecnica delle Marche, 60131 Ancona, Italy; m.caragiuli@univpm.it (M.C.); m.mandolini@staff.univpm.it (M.M.); d.landi@staff.univpm.it (D.L.)

**Keywords:** obstructive sleep apnea syndrome, mandibular advancement device, computer-aided design, digital workflow, digital dentistry

## Abstract

Background: Sleep-Related Breathing Disorders are characterized by repeated episodes of complete or partial obstruction of the upper airway during sleep. Mandibular advancement devices represent a non-invasive treatment in reducing the number of respiratory events and in decreasing symptoms. The advancement extent of these devices is responsible for the mandibular roto-translation and its effects on the temporomandibular joint. Methods: This study defined a systematic method to assess the mandible roto translation that is caused by MADs according to a scan-to-CAD approach. Starting from a closed mouth position and simulating the oral appliance at different settings it was possible to define a local reference system that is useful for the evaluation of the mandibular roto-translation. This latter was then applied to evaluate the movements of the condyle and the mandibular dental arch. Results: MAD1 resulted in a reduced mouth opening and protrusion, while MAD2 enabled a higher degree of motion of the mandible useful for patients who need an important protrusion. Conclusions: The two devices present different dynamics. Results that are achievable employing this method can be directly used by practitioners in comparing MADs, as well as by researchers in evaluating MADs effects.

## 1. Introduction

Sleep-Related Breathing Disorders are characterized by repeated episodes of complete (apnea) or partial (hypopnea/respiratory effort-related arousals) obstruction of the upper airway during sleep [1]. Obstructive sleep apnea (OSA) might affect the general health of a large percentage of the population, with an increasing prevalence in the last years of approximately 6% for men and 4% for women [2,3,4,5]. The diagnostic pathway of OSA starts from an anamnestic and clinical examination of the patient, followed by an instrumental evaluation through ambulatory or domiciliary polysomnography. The most frequent clinical symptoms are snoring, excessive daytime sleepiness and fatigue. These sleep changes can lead to the onset of important systemic dysfunctions, increasing the risk for cardiovascular and dysmetabolic diseases, and a significant reduction of the quality of life. 

The dentist expert in sleep medicine can actively contribute in the diagnosis and treatment of OSA. Oral appliances represent a recent non-invasive alternative treatment for OSA was demonstrated to be effective in reducing the number of respiratory events and decreasing symptoms [6,7,8]. The results obtained with mandibular advancement devices (MADs) are comparable to those obtained with the continuous positive airway pressure (CPAP), although the latter is more effective in reducing the number of apneas. The higher compliance rate and adherence to MADs therapy explain this result [9]. MADs are nocturnal intraoral appliances that stabilize the upper airways and increase their diameters, thus reducing pharynx collapsibility [10]. Xerostomia, headache, temporomandibular joint (TMJ) discomfort, dental soreness, and occlusal changes upon awakening are the most frequent short-term consequences. In the long term, there might be an overjet and overbite reduction and a decrease of posterior contacts [11,12,13].

To date, most of the studies concerning the effect of MAD treatment focus on upper airway investigation and occlusal changes. This study illustrates a systematic method for assessing the mandible roto-translation caused by MADs according to a scan-to-CAD approach. Applying this procedure can produce multiple advantages. From a clinical point of view, the advancement of a MAD is a central point for the therapy success and the development of complications in the long term [14]. Thus, it would be advantageous to predict the effect of the device on the condylar roto-translation to establish the optimal MAD settings. Standard dual-block titratable MADs are initially set at a percentage of maximum mandibular protrusion and progressively advanced to achieve during weeks the maximum advancement for normal breathing. The advantages of the proposed method are: (i) anticipated evaluation of the mandibular kinematic response before the treatment starts; and, (ii) prompt response of correction and optimization of the MAD settings. An improvement in the performance of the device would result in increased patient compliance and better treatment outcomes. Moreover, the effect of the MAD application can only be appreciated by analyzing the mandibular position while wearing the MAD and consequent further clinical examinations of the patient according to different methods. Polysomnography provides information on the apnea-hypopnea index (AHI), but null information on the TMJ kinematics. Several jaw-tracking methods [15] (optoelectronic, ultrasounds, and electromagnetic systems) provide a limited description of the mandibular position. Imaging techniques that are based on x-rays expose the patient to ionizing radiation and may present artifacts to the presence of metallic MADs components.

This paper aims to define a digital workflow, involving an optical 3D laser scanner, computer-aided design (CAD) software tools, dental plaster casts, and skull computed tomography (CT) images of patients, in order to evaluate the TMJ kinematic at different settings of the MAD. In particular, the paper presents: (i) a set of mandible and temporal bone landmarks and measurements for evaluating the mandibular and condylar kinematics, (ii) a digital workflow; and, (iii) its application on two MADs. This method avoids involving patients in further specific examinations as well as reduces the invasiveness on the jaw movement as much as possible. Besides the safety factor, a three-dimensional (3D) insight of the whole TMJ geometry enables the assessment of the mandibular position and dynamics about the temporal bone. Practitioners can benefit from this approach in comparing MADs according to the patient needs as well as researchers in evaluating MADs effects through numerical simulation. There are no previous works in the literature with this goal. 

## 2. Materials and Methods

The 3D model of the TMJ was reconstructed starting from the CT images (Voxel size 75 micron, FOV 11×13 cm. NewTom Giano, Cefla, Italy) using the software Mimics (v.12.11, Materialise NV, Leuven, Belgium). The CT sections were processed and investigated through computer-aided design software (Rhinoceros 3D by McNeel & Associates, Seattle, WA, USA). 

**Figure 1:**
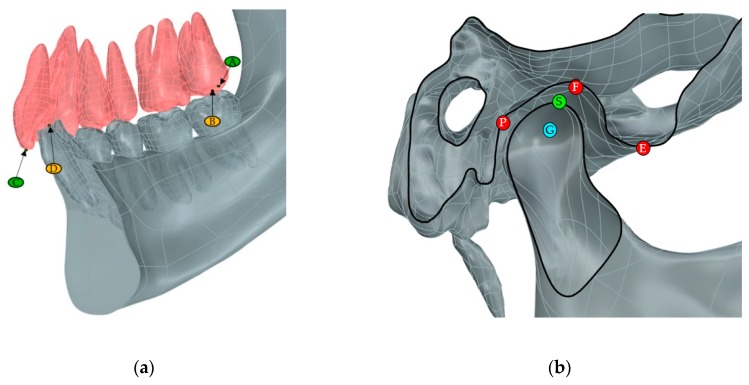
Mandible (**a**) and temporomandibular joint (TMJ) (**b**) landmarks in the section view.

The reconstructed model of the skull and the TMJ was oriented to reference planes, as reported in a previous study [16]. The sagittal plane perpendicular to the Frankfort horizontal plane divides the skull into symmetrical halves, thus all of the measurements to assess the mandibular displacement are taken in a section view according to this plane. The planar movements of the mandible are the result of combined rotation and translation (protrusion and opening) starting from a naturally closed mouth. Its kinematics can be evaluated in terms of Euclidean distances between anatomical landmarks localized while using anatomy manuals and atlas [17]. Table 1 reports a comprehensive description of them and Figure 1a,b provide a graphical representation.

The following sections describe the reference systems defined to assess the roto translations that are induced by the MADs over the mandible and all of the measurements required to evaluate the inter-jaws relationship and the condylar displacement and trajectory during the mandibular advancement. 

### 2.1. Mandibular Reference System and Measurements

**Figure 2:**
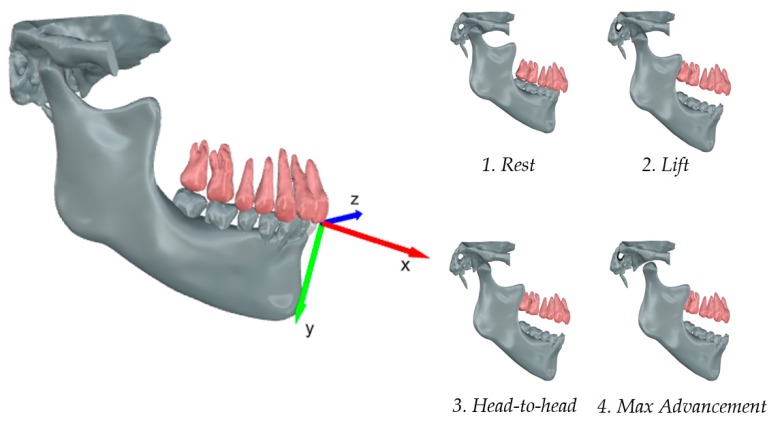
Local reference system (LRF) relative to the mandibular dynamics. The mandibular roto translation is displayed as four sequential configurations: Rest (1), Lift (2), Head-to-head (3), and Max advancement (4).

The inter-jaws relationship was assessed according to a local reference frame (LRF) that was centered between the tips of the left and right maxillary central incisors at the intersection with the sagittal plane (Figure 2).

The following procedure identified the reference frame. The authors set four anatomical landmarks in correspondence of the left and right maxillary molar distal cusps and the left and right maxillary incisal edges. Subsequently, the segments between contralateral molars and incisors landmarks intersected the sagittal plane in two points, being finally connected to form the *x*-axis. The anterior direction was assumed to be positive to describe the mandibular protrusion. The *z*-axis was perpendicular to the sagittal plane. The cross product of z and *x*-axis gave the *y*-axis, assumed as being positive downward according to the opening of the mandible. The symmetry to the sagittal plane allowed for only considering the half side of the jaw and teeth. The mandible was modeled with three degrees of freedom, allowing for translation on the XY plane and only a rotation around the *z*-axis constraining the model to a bidimensional plane. Positive rotations induced the mandible in a forward and downward position. 

The mandibular kinematics was evaluated in terms of protrusion and opening concerning the LRF, assuming the maxilla as being fixed. The horizontal movement of the mandible along the *x*-axis caused by the MAD incremental advancement measured the mandibular protrusion. The vertical displacement along the y-axis measured the mouth opening. Four configurations were analyzed.

- (Configuration 1) Rest referred to a naturally closed mouth without the MAD.
- (Configuration 2) Lift referred to the insertion of the MAD at minimum settings.
- (Configuration 3) Head-to-head referred to an edge-to-edge condition between the upper and lower incisors.
- (Configuration 4) Max advancement referred to the protrusion of the mandible due to the maximum settings of the MAD.

Two sets of measurements identified the distance between the upper and lower arches.

- Inter-molars between the upper and lower molar cusps (A–B distance, Figure 1a)
- Inter-incisors between the upper and lower incisors ridges (C–D distance, Figure 1a).

The former provides information on the thickness of the device during its introduction (Lift). The latter furnishes a tool to assess the incremental and total movement of the mandible at each configuration, starting from the resting state. The positive values refer to an anterior (along the *x*-axis) and inferior (along the *y*-axis) position of the mandible concerning the maxilla.

### 2.2. Condylar Displacement Characteristic Measurements

The relative motion of the condyle and the temporal bone was accomplished accordingly to Muto et al. [18]. The anatomical landmarks referred to a sagittal section plane (CP) passing from the midpoint of the line connecting the most medial and most lateral poles of the condyle in frontal view. Subsequently, the authors measured on this sagittal image the length (l) and depth (d) of the glenoid fossa, the minimum distance between the superior most point of the condyle and the temporal bone (ai), the minimum deviation of the condylar head from the temporal bone (bi), the condylar forward (ei), and inferior (fi) displacement from the articular eminence during the advancement of the mandible.

**Figure 3:**
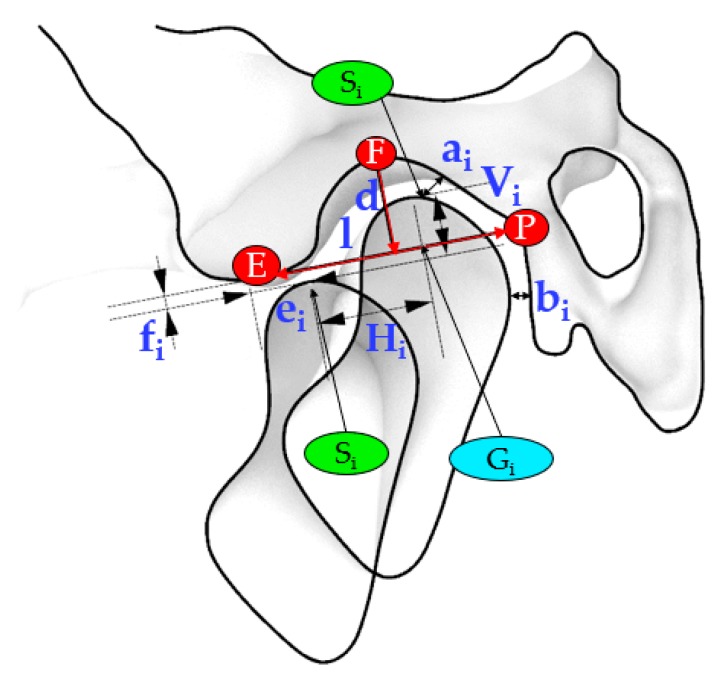
The central section (CP) shows the main relevant measurements to evaluate the movement of the condyle during the mandibular advancement.

The position of the condyle during the roto-translation was evaluated concerning the resting state. Therefore, it was possible to assess the distance between the S_i_ point at rest and during the mandibular movement according to the successive three configurations (S_2_, S_3_, S_4_), in terms of horizontal (H) and vertical (V) displacements. Figure 3 shows the sections that were used to investigate the relationship between the temporal bone and the mandibular condylar process according to the outlined distances.

Note that the measurements involved in the relationship between the condyle and the temporal bone (a_i_, b_i_) provided a quantitative evaluation of the position of the condyle concerning the glenoid fossa at Rest and its relationship with the articular eminence in correspondence with the successive configurations. 

Table 2 lists the measurements involved in the investigation of the relationship between the temporal bone and the condyle during mandibular advancement.

All of the measurements were taken when considering the temporal bone fixed. Thus, positive values refer to a forward and downward movement of the condyle from the landmarks located on the temporal bone or identifying a resting state configuration.

### 2.3. Workflow for Analyzing Mandible Measurements and Condyle Path

**Figure 4:**
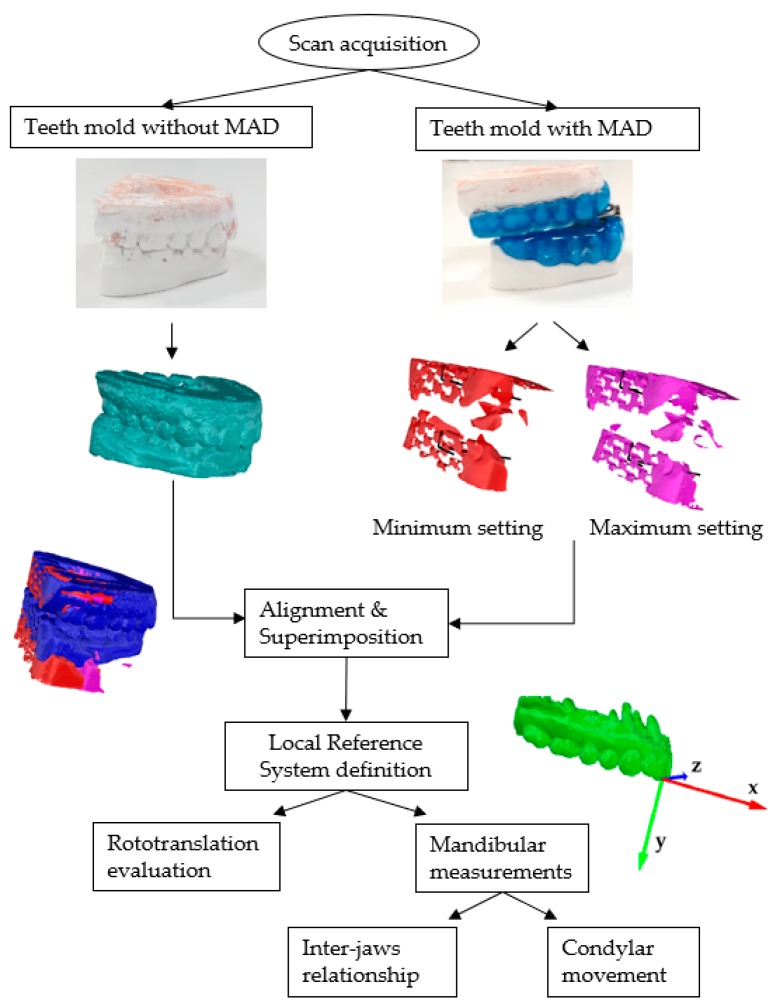
Workflow representing the main steps involved in the assessment of the mandibular kinematics when using a mandibular advancement device (MAD).

A scan-to-CAD approach was necessary for establishing the roto-translation induced by MAD at the four configurations. Software programs included Konica Minolta Range 7 as a non-contact 3D digitizer system, Range Viewer for reconstructing and aligning the surfaces, and Rhinoceros for 3D modeling. The workflow that is described in Figure 4 represents the steps to fulfill the aim of this study. 

**Figure 5:**
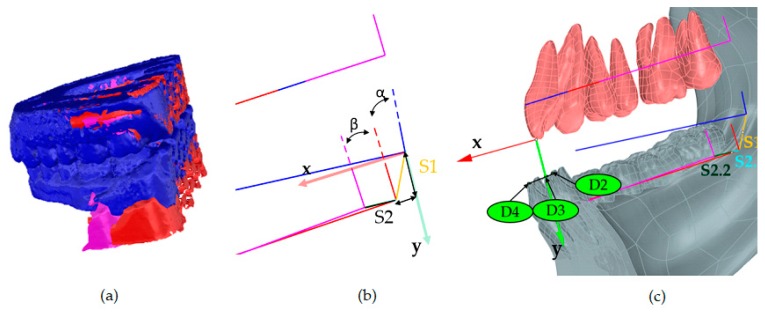
(**a**) Superimposition of the three scans of the dental casts at Rest (blue), Lift (red), Max advancement (magenta); (**b**) the construction reference frames (CRFs) overlapped in the upper arch provide the combined translation and rotation during Lift (S_1_),) and up to Max advancement (S_2_); (**c**) mandibular displacement due to the S_1_ and S_2_ roto translations and qualitative assessment of the Head-to-head configuration (S_2.1_).

The first step consisted in scanning the upper and lower dental casts in the resting state (configuration 1). Subsequently, after introducing the MAD (configuration 2), the authors scanned again the upper and lower dental casts. An additional scan (configuration 4) complete the set of scans. For evaluating the mandibular kinematics, the authors defined and realized a construction reference frame (CRF) made of three orthogonal vectors on the posterior surface of both the superior and inferior dental arches. The overlapping and successive matching of the three CRFs of the upper arch provides the proper configuration to evaluate the translation and the rotation of the mandible (Figure 5a,b). The colored lines, gold (S_1_) and black (S_2_), stand for a first roto-translation of the mandible after the introduction of a MAD at minimum setting (Lift) and a second roto-translation of the mandible to reach Max Advancement, respectively.

Quantitative assessment of mandible roto-translation during the four configurations is performed according to the LRF. The angle α between the CRF at rest and lift and the β angle between the axes of the CRF at Lift and Max Advancement measured the degree of rotation (Figure 5b). Positive rotations induce the mandible in a forward and downward position. The parameters (translation and rotation) that are related to Head-to-head configuration are retrieved starting from the previous information (Figure 5c). The mandible moved along S1 and S2 to track the corresponding position of the mandibular incisors, D2 and D4, respectively. The intersection between a line connecting the two previous points and the y-axis of the LRF provides the point D3 related to Head-to-head, since it makes null the horizontal distance between the maxillary and mandibular central incisors. A linear proportion between this segment and S2 allowed for us to retrieve the D3 point on S2, thus the S2.1 segment and successively to compute the rotation angle γ concerning Head-to-head. The difference between the angles β and γ measured the rotation of the mandible during the advancement from Head-to-head to Max Advancement. The length of the horizontal and vertical projections of S1, S2.1, and S2.2 on the LRF measured the amount of mandibular protrusion and opening. 

### 2.4. Method Testing

The analysis method was tested on a real OSA patient, which was asymptomatic for temporomandibular joint disorders. She gave the consent to use her skull CT scan and dental casts anonymously.

The oral appliances that were evaluated in this study were two custom-made adjustable mandibular advancement devices that covered the occlusal surfaces of the maxillary and mandibular teeth. Thus, all of the results are specific to this patient and not applicable for a general comparison.

**Figure 6:**
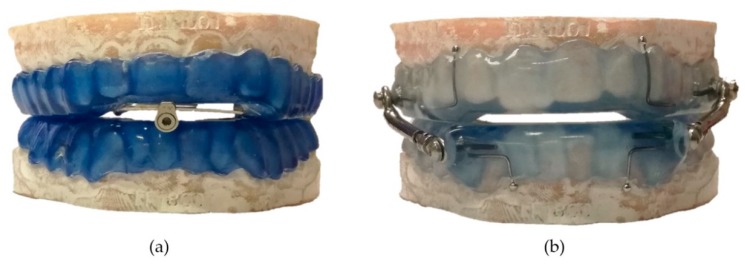
Mandibular advancement devices used in the present study: (**a**) MAD1 and (**b**) MAD2.

The initial setting of the oral appliances was at 70% of maximum mandibular protrusion determined by a George-Gauge fork. OrthoApnea (MAD1) (Figure 6a) consists of two splints that are joined by a central screw and two crossbars to allow for a millimetric-controlled advancement of the mandible modifying the permeability of the upper airway. The inverse connecting rod system permits the opening of the mandible and some lateral movement. Herbst (MAD2) (Figure 6b) has two acrylic-made splints that are joined by two stainless-steel lateral telescopic arms located at the molar-premolar region. The paired protrusion pins can slide in their respective guide tubes to protrude the mandible in a controlled incremental advancement.

## 3. Results

Table 3 summarizes the measurements of the mandibular roto-translation in terms of protrusion, opening, and rotation corresponding to three sequential configurations (Lift, Head-to-head, and Max Advancement).

Table 4 reports the inter-jaws relationship during the mandibular motion by evaluating the distances between the maxillary and mandibular teeth (both incisors and molars) in correspondence with the four configurations, assuming the maxilla as being fixed. Further information is provided by the relative relationship between the mandibular incisors during the movement.

The investigation of the condylar process displacement concerning the temporal bone provided the results that are listed in Table 5.

**Figure 7:**
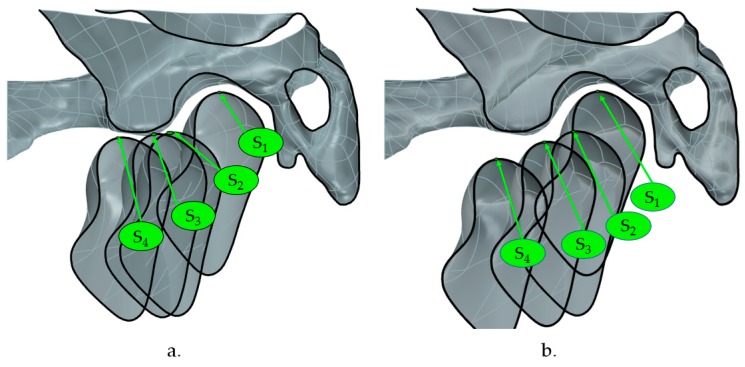
The total condylar trajectory of MAD1 (**a**) and MAD2 (**b**) in correspondence of the following configurations: Rest, Lift, Head-to-head, and Max Advancement.

Figure 7 shows a simulation of the mandibular movement induced by MADs focusing attention on the condylar trajectory concerning the temporal bone assumed as being fixed.

## 4. Discussion

### 4.1. Digital Workflow Performance

Several methods measure mandibular kinematics [19]. Some rely on mechanical devices, such as articulators and axiographs, which do not provide any morphological characterization of the TMJ, but only a qualitative investigation of the condylar motion. Others attempt to digitally track the mandibular motion by employing different sensing elements, such as light-emitting diodes magnetometers and ultrasounds. Optoelectronic systems are the most common, since they do not require particular skills, only a complex camera setting and a well-controlled environment. However, these instruments, applied to the patient’s dental arch, merely evaluate the position of the teeth after a precise mandibular movement. X-rays-based methods (lateral radiograph, CT, and videofluoroscopy) provide precise two-dimensional (2D) or 3D anatomical information on mandibular motion, but expose patients to harmful ionizing radiation. Most of the studies that involve MAD therapy in OSA provide evidence of the occlusal changes after a long-term treatment by clinical examination through sliding calipers or cephalography and information on the airway flow improvement through PSG evaluation. 

In contrast, the current approach outlines a method that is useful to characterize the mandibular kinematics concerning the temporal bone by reproducing the whole TMJ geometry under the effect of MAD. The functional activity of the MAD is performed on dental plaster casts and evaluated through a highly accurate laser scanner while using 3D modeling software without interfering with the mandibular motion. For this reason, it is also very flexible and adaptable to different types of MADs. The mandibular roto translation assessment might be evaluated in the 3D space or a sagittal plane and enables examining the inter-jaws relationship and the condylar displacement and rotation concerning the temporal bone. We could also estimate the behavior of the condyle according to a precise type of MAD and patient to improve the MAD settings for optimal treatment. Besides this, the clinician will be able to opt for the best treatment solution when comparing the predicted mandibular position according to different types of MADs. All of the measurements performed in a virtual environment do not involve the patient, resulting in almost null invasiveness apart from the preliminary CT scan that is required for the TMJ clinical examination.

Despite this, data processing requires different software tools, significant operational time, a skilled operator, and high equipment costs. A further improvement can involve the automation of the post-processing to speed-up the workflow.

### 4.2. TMJ Kinematics Findings

The evaluation of the inter-jaws relationship that is associated with wearing a MAD revealed that the simple introduction of the MAD in a naturally closed mouth induces a mandibular rotation resulting in a pronounced mouth opening, partially due to the thickness of the splints of the oral device. From Table 3, note that the incremental advancement of the MAD is associated with a small rotation angle, which contributes to a mouth opening, decreasing the efficacy of the treatment, as reported by Milano et al. [20]. There is a difference in mouth opening between the two devices evaluated. MAD 1 determines a lower mouth opening, as reported in Table 3 concerning the MAD 2, but consider that the maximum protrusion of the MAD 1 is lower than the MAD 2. Table 4 permits evaluating whether the two devices have a similar posterior opening (distance A-B2 is 9 mm for the MAD 1 and 8.9 mm for the MAD 2). Another difference between the devices in the lift position is that MAD 1 presents a significant protrusion of 5.6 mm, while the MAD 2 protrudes 3 mm. On the other hand, the MAD 2 allows for a higher final protrusion during the titration protocol. A MAD that induces a more closed mandibular position and it does not permit to open the mouth should be preferred unless the patient cannot tolerate this forced position. Alternatively, intermaxillary elastics can help in closing the mouth in those MADs that allow for the mouth opening.

The measurements at Max Advancement highlighted how the condyle moved far beyond the summit of the articular eminence, resulting in a mandibular protrusion of approximately 11.65 mm ± 1.55mm (averaged value of D1-D4 Table 4) from resting state. Muto et al. [18] found out a significant correlation between the forward condylar translation from the eminence and increased mouth opening. Figure 7 showed different condylar kinematics, with the MAD 1 determining a closer position of the condyle to the temporal bone during the trajectory. A clinician should avoid inducing a disc compression or dislocation in patients that are affected by temporomandibular disorders. The differences in mouth opening between the two devices can influence the therapeutic choice. MAD 1, with its higher protrusion in lift position, could be the best treatment solution for patients that require an immediate response to the therapy. On the other side, the choice of MAD 2 can be better suited for the patients that need a consistent advancement to solve their condition. However, in the every-day practice, it is useful to customize on the patient’s clinical condition and his preference for an appliance, since the compliance rate influences the outcome of our intervention.

This study also presents some limitations concerning the achieved results. Indeed, the data obtained are model-dependent and not applicable for every patient due to individual anatomical differences, craniofacial morphologies, and masticatory muscle activities. Different titration protocols of the device can also influence treatment results. Nevertheless, the procedure is reliable and applicable to every MAD to examine the mandibular roto-translation and the effects on TMJ.

For overcoming above mentioned limitations, it is expected, as future work, to characterize other combinations of MADs (i.e., different for mechanism) and patients (i.e., different for AHI). The investigation of the latter is required for evaluating the effects of different cranio-facial morphologies. At last, the dataset of these results can be used for analyzing loads, stresses, and deformations of temporomandibular joints (i.e., muscles, articular disc, joint capsule, mandible, and temporal bone), by employing finite element methods (FEM) and/or multibody (MB) approaches [21,22,23,24]. Results that are achievable by using this digital workflow represent the boundary conditions of FEM and MB models. 

## 5. Conclusions

The two devices present different dynamics regarding mouth opening, maximum protrusion, and protrusion during advancement. On the other side, the devices show a similar posterior opening. The simulation of the mandibular movement that is induced by MADs shows the different trajectory of the condyle, with a possible increased articular disc compression in MAD 1 than MAD 2. Further FEM and clinical studies should focus on the MAD effects on the temporomandibular joint.

## Figures and Tables

**Table 1:** Landmarks used in this study.

Landmark	Definition
A	Distal cusp tip of the second maxillary molar (Figure 1a)
B	Distal cusp tip of the second mandibular molar (Figure 1a)
C	Point of intersection between the sagittal plane and the line connecting the tips of the maxillary central incisors (left and right) (Figure 1a)
D	Point of intersection between the sagittal plane and the line connecting the tips of the mandibular central incisors (left and right) (Figure 1a)
E	The most caudal point of the articular eminence (Figure 1b)
F	The point on a tangent to the most superior aspect of the glenoid fossa parallel to the P-E line that intersects the fossa (Figure 1b)
G	The midpoint of the line intersecting the posterior and anterior margins of the condyle when a reference line is drawn from point P to point E (Figure 1b)
P	The point of the apex of post glenoid spine (Figure 1b)
S	Superior-most point of the condyle (Figure 1b)

**Table 2:** Main relevant measurements that are useful for investigating the TMJ kinematics. * The subscript number refers to the i-th configuration.

Measurements *	Definition
d	Depth of the glenoid fossa: the distance between F point and the point at which the P-E line is intersected by the perpendicular line constructed from F point to the P-E line
l	Length of the glenoid fossa: the distance between P point and E point
ai	Minimum distance between the uppermost point of the condyle (Si) and the temporal bone
bi	Minimum deviation of the head of the condyle from the temporal bone
ei	Forward condylar displacement: the distance E-Gi between the articular eminence (E) and the apex of the condyle (Gi) parallel to the P-E line
fi	Inferior condylar displacement: the distance between Gi point and the point at which the P-E line is intersected by the perpendicular line constructed from Gi point to the P-E line
Hi	The distance between the uppermost point of the condyle (Si) and the uppermost point of the condyle at Rest, projected on the P-E line
Vi	The distance between the uppermost point of the condyle (Si) and the uppermost point of the condyle at Rest, projected on the perpendicular to the P-E line

**Table 3:** Mandibular roto translations (vertical opening, horizontal protrusion, and rotation angle) induced by MADs.

	MAD1			MAD2		
Configurations	Opening [mm]	Protrusion [mm]	Rotation [degree°]	Opening [mm]	Protrusion [mm]	Rotation [degree°]
Lift	7.4	3.7	5.97	7.7	1.1	4.89
Head-to-head	−0.1	1.8	0.44	0.1	4.4	−0.35
Max advancement	−0.2	4.1	1.01	0.2	7.3	−0.58
Total	7.1	9.6	7.42	8.0	12.8	3.96

**Table 4:** Assessment of inter-jaws dynamics from resting state to mandibular maximum advancement. Distances are evaluated concerning the resting state (configuration 1) and concerning the previous configuration (configuration i-1).

		MAD1		MAD2	
Configurations	Distances	Opening [mm]	Protrusion [mm]	Opening [mm]	Protrusion [mm]
**Resting state**	C_1_-D_1_	−3.7	−6.0	−3.7	−6.0
	A_1_-B_1_	0.6	1.5	0.6	1.5
**Lift**	C_1_-D_2_	8.3	−1.8	7.6	−4.4
	A_1_-B_2_	9.0	5.6	8.9	3.0
	D_1_-D_2_	12.0	4.2	11.3	1.6
**Head-to-head**	C_1_-D_3_	8.5	0.0	7.5	0.0
	D_1_-D_3_	12.2	6.0	11.2	6.0
	D_2_-D_3_	0.2	1.8	−0.1	4.4
**Max Advancement**	C_1_-D_4_	9.1	4.2	7.2	7.2
	D_1_-D_4_	12.8	10.1	11.0	13.2
	D_3_-D_4_	0.6	4.2	−0.2	7.2

**Table 5:** Evaluation of the condylar displacement under the usage of the MADs. * The subscript number refers to the i-th configuration.

		MAD1	MAD2
Configurations	Distances [mm] *	CP	CP
**Rest**	d	6.3	6.3
	l	18.0	18.0
	a_1_	2.2	2.2
	b_1_	1.4	1.4
**Lift**	a_2_	1.4	3.7
	b_2_	1.2	3.0
	e_2_	−3.9	−7.2
	f_2_	0.9	1.4
	H2	7.6	4.2
	V2	4.2	4.7
**Head-to-head**	a_3_	0.6	2.3
	b_3_	0.6	2.2
	e_3_	−1.8	−3.1
	f_3_	0.7	2.1
	H3	9.8	8.4
	V3	4.0	5.4
**Max advancement**	a_4_	1.2	4.0
	b_4_	0.8	3.9
	e_4_	3.1	3.7
	f_4_	0.2	3.2
	H4	14.7	15.1
	V4	3.5	6.5
